# Supplementary material for: Empirical methods for controlling false positives and estimating confidence in ChIP-Seq peaks
Source: BMC Bioinformatics. 2008 Dec 5;9:523. doi: 10.1186/1471-2105-9-523 (PMC2628906; doi:10.1186/1471-2105-9-523)
Supplement: Additional File 1 — A variety of html documents from the USeq web site detailing the available applications, their best usage, output file type descriptions, command line menus, etc. [file 1471-2105-9-523-S1.zip › USeqUserGuides/JavaDoc.zip!/JavaDoc/bioroot/ReagentBean.html]

ReagentBean


|  |  |  |
| --- | --- | --- |
| **Overview** \| **Package** \| **Class** \| **Use** \| **Tree** \| **Deprecated** \| **Index** \| **Help** | |  |
| **PREV CLASS**   **NEXT CLASS** | **FRAMES**    **NO FRAMES**     **All Classes** |  |
| SUMMARY: NESTED \| FIELD \| CONSTR \| METHOD | DETAIL: FIELD \| CONSTR \| METHOD |  |


---


## bioroot Class ReagentBean

```
java.lang.Object
  bioroot.ReagentBean
```

**All Implemented Interfaces:**: java.lang.Comparable

**Direct Known Subclasses:**: AntibodyBean, OligoBean, PlasmidBean, StrainBean

---

``` public class ReagentBean extends java.lang.Object implements java.lang.Comparable ```

**Author:**
:   nix
    Container for holding comon information about a reagent (oligo, plasmid, strain, antibody...)

---

| **Field Summary** | |
| --- | --- |
| `static org.apache.log4j.Logger` | `log` |


**Constructor Summary**

`ReagentBean()`


| **Method Summary** | |
| --- | --- |
| `void` | `appendMessages(java.lang.String string)` |
| `boolean` | `canModifyAll(UserBean user)` |
| `boolean` | `canModifyCommon(UserBean user)` |
| `boolean` | `canView(UserBean user, DBUtil bioRoot)` |
| `int` | `compareTo(java.lang.Object obj)` |
| `java.lang.String` | `getAlias()` |
| `java.lang.String` | `getAvailability()` |
| `java.lang.String` | `getComments()` |
| `java.lang.String` | `getEditHistory()` |
| `java.lang.String` | `getGenericVisibility()`             Returns one of the standard visibility settings (WWW, Lab mates, Private) or the generic 'Organization' |
| `int` | `getId()` |
| `int` | `getLabGroupId()` |
| `java.lang.String` | `getLabGroupName(DBUtil bioRoot)` |
| `java.lang.String` | `getLastUser()` |
| `java.lang.String` | `getLocation()` |
| `java.lang.String` | `getMessages()` |
| `java.lang.String` | `getName()` |
| `java.lang.String` | `getNotes()` |
| `java.lang.String` | `getOrderBy()` |
| `int` | `getOrganismId()` |
| `java.lang.String` | `getOrganismName(DBUtil bioRoot)` |
| `Organization` | `getOrganization(DBUtil bioRoot)` |
| `int` | `getOwnerId()` |
| `java.lang.String` | `getOwnerName(DBUtil bioRoot)` |
| `java.lang.String` | `getReference()` |
| `java.lang.String` | `getVisibility(DBUtil bioRoot)` |
| `boolean` | `isComplete()` |
| `boolean` | `isReagentNameUnique(DBUtil bioRoot, java.lang.String tableName)`             checks to see if oligo name is unique within labgroup, assumes a connection has been made |
| `void` | `loadReagentBean(java.sql.ResultSet results)` |
| `void` | `setAlias(java.lang.String string)` |
| `void` | `setAvailability(java.lang.String string)` |
| `void` | `setComments(java.lang.String comments)` |
| `void` | `setComplete(boolean complete)` |
| `void` | `setEditHistory(java.lang.String editHistory)` |
| `void` | `setId(int id)` |
| `void` | `setLabGroupId(int labGroupId)` |
| `void` | `setLabGroupName(java.lang.String labGroupName)` |
| `void` | `setLastUser(java.lang.String lastUser)` |
| `void` | `setLocation(java.lang.String string)` |
| `void` | `setMessages(java.lang.String string)` |
| `void` | `setName(java.lang.String string)` |
| `void` | `setNotes(java.lang.String string)` |
| `void` | `setOrderBy(java.lang.String orderBy)` |
| `void` | `setOrganismId(int i)` |
| `void` | `setOrganismName(java.lang.String organismName)` |
| `void` | `setOwnerId(int i)` |
| `void` | `setOwnerName(java.lang.String ownerName)` |
| `void` | `setReference(java.lang.String string)` |
| `void` | `setVisibility(java.lang.String string)` |
| `boolean` | `updateCommonAccessFields(DBUtil bioRoot, java.lang.String reagentType)`             Just updates fields that are accessible by all lab group memebers, lastUser, comments given a reagent type: Oligo or Plasmid. |

**Methods inherited from class java.lang.Object**

`equals, getClass, hashCode, notify, notifyAll, toString, wait, wait, wait`

**Field Detail**

### log

```
public static org.apache.log4j.Logger log
```


**Constructor Detail**

### ReagentBean

```
public ReagentBean()
```


**Method Detail**

### compareTo

```
public int compareTo(java.lang.Object obj)
```

:   **Specified by:**: `compareTo` in interface `java.lang.Comparable`

---


### loadReagentBean

```
public void loadReagentBean(java.sql.ResultSet results)
```

---


### updateCommonAccessFields

```
public boolean updateCommonAccessFields(DBUtil bioRoot,
                                        java.lang.String reagentType)
```

:   Just updates fields that are accessible by all lab group memebers, lastUser, comments given a
    reagent type: Oligo or Plasmid. Strain overrides this method.

---


### isReagentNameUnique

```
public boolean isReagentNameUnique(DBUtil bioRoot,
                                   java.lang.String tableName)
```

:   checks to see if oligo name is unique within labgroup, assumes a connection has been made

---


### canView

```
public boolean canView(UserBean user,
                       DBUtil bioRoot)
```

---


### canModifyAll

```
public boolean canModifyAll(UserBean user)
```

---


### canModifyCommon

```
public boolean canModifyCommon(UserBean user)
```

---


### getVisibility

```
public java.lang.String getVisibility(DBUtil bioRoot)
```

---


### getGenericVisibility

```
public java.lang.String getGenericVisibility()
```

:   Returns one of the standard visibility settings (WWW, Lab mates, Private) or the generic 'Organization'

---


### getLabGroupName

```
public java.lang.String getLabGroupName(DBUtil bioRoot)
```

---


### getOwnerName

```
public java.lang.String getOwnerName(DBUtil bioRoot)
```

---


### getOrganismName

```
public java.lang.String getOrganismName(DBUtil bioRoot)
```

---


### getOrganismId

```
public int getOrganismId()
```

---


### getAlias

```
public java.lang.String getAlias()
```

---


### getAvailability

```
public java.lang.String getAvailability()
```

---


### getEditHistory

```
public java.lang.String getEditHistory()
```

---


### getId

```
public int getId()
```

---


### getLabGroupId

```
public int getLabGroupId()
```

---


### getLocation

```
public java.lang.String getLocation()
```

---


### getMessages

```
public java.lang.String getMessages()
```

---


### getName

```
public java.lang.String getName()
```

---


### getNotes

```
public java.lang.String getNotes()
```

---


### getOwnerId

```
public int getOwnerId()
```

---


### isComplete

```
public boolean isComplete()
```

---


### getOrderBy

```
public java.lang.String getOrderBy()
```

---


### setAlias

```
public void setAlias(java.lang.String string)
```

---


### setAvailability

```
public void setAvailability(java.lang.String string)
```

---


### setLocation

```
public void setLocation(java.lang.String string)
```

---


### setMessages

```
public void setMessages(java.lang.String string)
```

---


### appendMessages

```
public void appendMessages(java.lang.String string)
```

---


### setName

```
public void setName(java.lang.String string)
```

---


### setNotes

```
public void setNotes(java.lang.String string)
```

---


### setOrganismId

```
public void setOrganismId(int i)
```

---


### setOwnerId

```
public void setOwnerId(int i)
```

---


### setVisibility

```
public void setVisibility(java.lang.String string)
```

---


### setComplete

```
public void setComplete(boolean complete)
```

---


### setEditHistory

```
public void setEditHistory(java.lang.String editHistory)
```

---


### setId

```
public void setId(int id)
```

---


### setLabGroupId

```
public void setLabGroupId(int labGroupId)
```

---


### setOrderBy

```
public void setOrderBy(java.lang.String orderBy)
```

---


### setLabGroupName

```
public void setLabGroupName(java.lang.String labGroupName)
```

---


### setOrganismName

```
public void setOrganismName(java.lang.String organismName)
```

---


### setOwnerName

```
public void setOwnerName(java.lang.String ownerName)
```

---


### getComments

```
public java.lang.String getComments()
```

---


### setComments

```
public void setComments(java.lang.String comments)
```

---


### getLastUser

```
public java.lang.String getLastUser()
```

---


### setLastUser

```
public void setLastUser(java.lang.String lastUser)
```

---


### getReference

```
public java.lang.String getReference()
```

---


### setReference

```
public void setReference(java.lang.String string)
```

---


### getOrganization

```
public Organization getOrganization(DBUtil bioRoot)
```


---


|  |  |  |
| --- | --- | --- |
| **Overview** \| **Package** \| **Class** \| **Use** \| **Tree** \| **Deprecated** \| **Index** \| **Help** | |  |
| **PREV CLASS**   **NEXT CLASS** | **FRAMES**    **NO FRAMES**     **All Classes** |  |
| SUMMARY: NESTED \| FIELD \| CONSTR \| METHOD | DETAIL: FIELD \| CONSTR \| METHOD |  |


---
